# Predicting the genetic impact of stocking in Brook Charr (*Salvelinus fontinalis*) by combining RAD sequencing and modeling of explanatory variables

**DOI:** 10.1111/eva.12566

**Published:** 2017-11-13

**Authors:** Justine Létourneau, Anne‐Laure Ferchaud, Jérémy Le Luyer, Martin Laporte, Dany Garant, Louis Bernatchez

**Affiliations:** ^1^ Département de Biologie Institut de Biologie Intégrative et des Systèmes (IBIS) Université Laval Québec QC Canada; ^2^ Département de Biologie Faculté des Sciences Université de Sherbrooke Sherbrooke QC Canada

**Keywords:** Brook Charr, fishery management, genotyping by sequencing, introgression, stocking

## Abstract

In fisheries management, intensive stocking programs are commonly used to enhance population abundance and maintain stock productivity. However, such practices are increasingly raising concerns as multiple studies documented adverse genetic and evolutionary impacts of stocking on wild populations. Improvement of stocking management relies on a better understanding of the dynamic of introgressive hybridization between wild and domestic population and on assessment of the genetic state of wild populations after stocking cessation. In Québec, Canada, over five million captive‐reared Brook Charr (*Salvelinus fontinalis*) are stocked every year to support recreational fishing activities. Here, we investigated how variation in stocking history and environmental variables, including water temperature, pH, and dissolved oxygen, may influence the impact of stocking practices on the genetic integrity of wild Brook Charr populations. We collected DNA samples (*n* = 862, average of 30 individuals per lake) from 29 lakes that underwent different stocking intensity through time and also collected environmental parameters for each sampled lake. An average of 4,580 high‐quality filtered SNPs was obtained for each population using genotyping by sequencing (GBS), which were then used to quantify the mean domestic membership of each sampled population. An exhaustive process of model selection was conducted to obtain a best‐fitted model that explained 56% of the variance observed in mean domestic genetic membership. The number of years since the mean year of stocking was the best explanatory variable to predict variation in mean domestic genetic membership whereas environmental characteristics had little influence on observed patterns of admixture. Our model predictions also revealed that each sampled wild population could potentially return to a wild genetic state (absence of domestic genetic background) after stocking cessation. Overall, our study provides new insights on factors determining level of introgressive hybridization and suggests that stocking impacts could be reversible with time.

## 1 INTRODUCTION

Commercial and recreational exploitation of many wild fish populations has reached and even exceeded the threshold for maximum sustainable yield (Dunham, 2011). Many populations are showing important declines because of overfishing and environmental change (Allan et al., 2005; Dunham, 2011; Hoegh‐guldberg & Bruno, 2016). As a result, supplementation (hereafter stocking) programs based on releases of captive‐reared (domesticated) fish are now used worldwide to counteract the negative effects of overexploitation by increasing the absolute size of fish stocks (North American Commission, Nasco Scientific Working Group, 1992; Ritter, 1997). Yet, numerous studies have documented the potentially negative effects of stocking on the genetic integrity of wild populations as well as on their evolutionary potential (Ryman & Laikre, 1991; Rhymer & Simberloff, 1996; Laikre & Ryman, 1996; Araki, Cooper et al., 2007; Fraser, 2008; Araki, Berejikian, Ford, & Blouin, 2008; Laikre, Schwartz, Waples, & Ryman, 2010). Possible impacts of stocking include a significant decrease in effective population size, due to the low number of reproducers used to perform supportive breeding (Ryman & Laikre, 1991; Laikre & Ryman, 1996; Hansen, Nielsen, Ruzzante, Bouza, & Mensberg, 2000; Wang & Ryman, 2001; Laikre et al., 2010), a loss of genetic diversity in stocked populations (Eldridge, Myers, & Naish, 2009) and a loss of genetic differentiation between populations (Eldridge & Naish, 2007; Eldridge et al., 2009; Hansen, Fraser, Meier, & Mensberg, 2009; Marie, Bernatchez, & Garant, 2010; Lamaze, Sauvage, Marie, Garant, & Bernatchez, 2012; Perrier, Guyomard, Bagliniere, Nikolic, & Evanno, 2013).

The incorporation of alleles from a population into the gene pool of another genetically distinct populations, (e.g., introgressive hybridization), is another threat to the genetic integrity of stocked populations (Araguas, Sanz, Pla, & Garcia‐Marin, 2004; Hansen, Bekkevold, Jensen, Mensbergand, & Nielsen, 2006; Eldridge & Naish, 2007; Marie et al., 2010). Indeed, although hybridization is a natural process sometimes contributing to diversification and adaptability (Dowling & Secor, 1997; Allendorf, Leary, Spruell, & Wenburg, 2001), it can also lead to loss of genotypically different populations and increase extinction risk (Rhymer & Simberloff, 1996; Seehausen, Takimoto, Roy, & Jokela, 2008; Kelly, Whiteley, & Tallmon, 2010; Gozlan, Britton, Cowx, & Copp, 2010). In the case of stocking, as domestic fish and their wild counterparts undergo drastically different selection regimes, captive individuals often tend to do poorly in natural environment. (Laikre & Ryman, 1996; Ford, 2002; Fraser, 2008; Christie, Marine, French, & Blouin, 2012). Furthermore, in addition to domestication, genetic load due to inbreeding and relaxed sexual selection in captive stocks could also explain the lower fitness of domestic fish when released in the wild (Ford, 2002; Mcginnity et al., 2003; Araki, Ardren, Olsen, Cooper, & Blouin, 2007; Araki et al., 2008; Frankham, Ballou, & Briscoe, 2010; Christie et al., 2012). Therefore, reproduction between domestic and wild fish can result in the loss of local adaptation to the environmental conditions of wild populations (Mcginnity et al., 2003; Araki, Ardren et al., 2007; Araki et al., 2008 Finnegan & Stevens, 2008; Hansen et al., 2009) or the disruption of co‐adapted genes complex through introgression (Laikre & Ryman, 1996; Allendorf et al., 2001; Ford, 2002; Tallmon, Luikart, & Waples, 2004; Edmands, 2007; Laikre et al., 2010; Allendorf, Hohenlohe, & Luikart, 2010).

In salmonid fishes, several factors were also shown to affect the extent of introgressive hybridization between wild and domestic populations. Namely, it has been documented that admixture level tends to be correlated with stocking intensity in terms of number of fish stocked per hectare and/or the number of stocking events (Almodódovar, Nicola, Elvira, & Garcia‐Marín, 2006; Finnegan & Stevens, 2008; Hansen & Mensberg, 2009; Marie et al., 2010; Lamaze et al., 2012). However, the size of wild populations (Hansen et al., 2009; Perrier, Baglinière, & Evanno, 2012) and the survival and reproductive success of domestic fish (Araki et al., 2008) could also influence admixture rates independently from stocking intensity. Also, it has been suggested that time spent following stocking events may be an important factor influencing admixture proportion (Hansen & Mensberg, 2009; Hansen et al., 2009; Perrier et al., 2013; Valiquette, Perrier, Thibault, & Bernatchez, 2014; Harbicht, Wilson, & Fraser, 2014). For instance, after stocking cessation, the genetic background originating from the populations used for stocking tends to decrease with time and eventually almost disappears in Lake Trout (*Salvelinus namaycush*) populations in Québec, Canada (Valiquette et al., 2014).

The detection of introgressive hybridization between two different populations can be accomplished using few genetic markers but many markers are required to assess the proportion of admixture within individuals (Allendorf et al., 2010). Indeed, using a reduced number of markers can be misleading when hybridizing populations are closely related, as hybrids in those populations can be difficult to identify correctly (Hansen & Mensberg, 2009; Ozerov et al., 2016; Vähä & Primmer, 2006). Furthermore, differential rates of introgression among different genomic regions have also been documented, including in salmonid species (Lamaze et al., 2012; Ozerov et al., 2016). These observations emphasize the potential benefit of using a larger number of single nucleotide polymorphisms (SNPs) toward better understanding the dynamics of introgressive hybridization.

The Brook Charr is a salmonid species native from Eastern North America. It is widely distributed in Eastern Canada, and populations are found in clear and well‐oxygenated water of rivers and lakes (Scott & Crossman, 1973). In Québec, Canada, Brook Charr recreational fishing supports an industry generating 600 millions$ per year (Fisheries and Oceans Canada 2013). To support this economically important activity, intensive stocking programs have been conducted since 1970. Hence, every year, more than 650 tons of Brook Charr are stocked, which represents 70% of the annual production of Québec fish farming (*Ministère du Développement Durable, de l'Environnement, de la Faune et des Parcs* 2013 *)*. The strain of Brook Charr largely used in Québec for stocking originates from many crosses between two freshwater strains (Nashua and Baldwin) and has been cultivated for more than one hundred years. Many differences between domestic and wild strains have been documented over the years (Sauvage et al., 2010; Lamaze et al., 2012; Crespel, Bernatchez, Audet, & Garant, 2013a,b). For instance, Bougas, Granier, Audet, and Bernatchez (2010) observed that domestic and wild strains differed significantly in terms of the number and nature of differentially expressed genes in controlled conditions. Some of those differences could be the result of directional selection by fish farmers for traits of commercial interest such as growth, disease resistance, and swimming resistance. Stocking history of several lakes has been recorded by governmental institutions, thus providing an excellent context to study the influence of stocking intensity along with environmental variables on the extent of introgressive hybridization between wild and domestic populations. Indeed, a previous study on Brook Charr populations in Québec conducted by Marie et al. (2010); Marie, Bernatchez, Garant, and Taylor (2012) used microsatellite markers to assess the impact of stocking practices and environmental factors on hybridization level. In addition to observing the effect of intense stocking on the genetic structure of wild populations (Marie et al., 2010), they also showed that hybridization was pronounced in smaller and shallower lakes and increased with water temperature and pH, but decreased with dissolved oxygen (Marie et al., 2012; but see Harbicht, Alshamlih, Wilson, & Fraser, 2014). The results of Marie et al. (2012) suggest that less favorable environmental conditions for the species could increase the hybridization level. However, because of the limited temporal coverage of the number of years since the last stocking events, this study could not address the question pertaining to the potential resilience capacity of wild populations. Also, this study did not specifically aim at building a model able to predict the genetic impact of stocking using stocking intensity and environmental variables and relied on a relatively small number of genetic markers.

In this context, the ultimate purpose of this study was to develop a statistical model allowing the prediction of admixture proportion between wild and domestic populations of Brook Charr combining a set of variables describing the stocking history and environmental characteristics of each individual population being studied. We specifically aimed to (i) assess admixture proportion in stocked populations of Brook Charr using a genomewide approach, (ii) test and define a best‐fitted model able to explain observed variation in admixture proportions between wild and domestic populations sampled using stocking history and environmental variables, and to (iii) investigate the resilience capacity of populations by determining the number of years needed for the populations to go back to a state of origin after the stocking cessation.

## 2 MATERIAL AND METHODS

### 2.1 Sampling strategy

**Figure 1:**
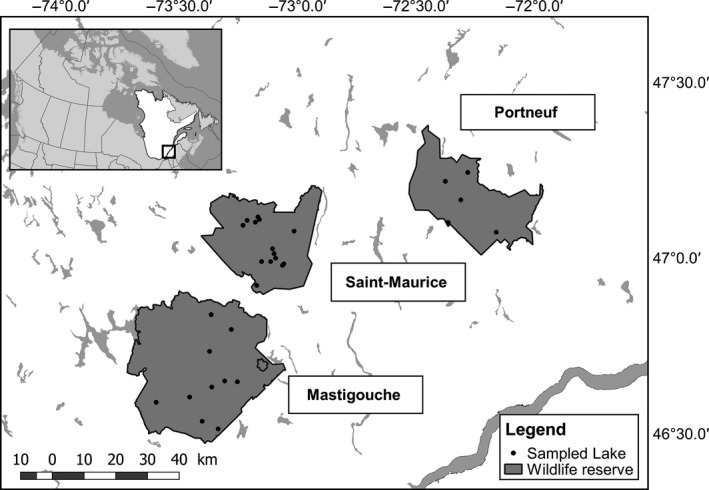
Geographical locations of sampled lakes in three wildlife reserves in the province of Québec, Canada, for this study on Brook Charr

Sampling was conducted in three different wildlife reserves (e.g., Portneuf, Mastigouche, and St‐Maurice) in Québec, Canada, which were created in 1971 and where fishing is strictly regulated and managed. Stockings were used in many lakes at various intensities over time to support angling and to reduce fishing pressure on natural populations and the history of stockings has been well recorded since the creation of the reserves. Therefore, we selected 29 lakes according to their different stocking histories representing a continuum of diverse stocking intensities based on (i) the stocking frequency, (ii) the year of the last stocking event, and (iii) the quantity of stocked fish. Five lakes were sampled in Portneuf, ten in Mastigouche, and fourteen in Saint‐Maurice Reserve, respectively (Figure 1). A total of 862 Brook Charr (from 21 to 45 individuals per lake, mean = 30) were sampled in summer (June to August) 2014 and 2015 using experimental gillnets with different mesh sizes (Table 1). The stocked Brook Charr used to supply the selected lakes came from different hatcheries: fish originated from *Jacques‐Cartier* hatchery for the Portneuf Reserve and *Truites de la Mauricie* Aquaculture Center for the Mastigouche and Saint‐Maurice reserves. It is noteworthy that domestic fish from the different hatcheries all share the same origin and are genetically very similar (Martin, Savaria, Audet, & Bernatchez, 1997). Fin tissues from 91 individuals were obtained from these two domestic strains (56 individuals and 35 individuals, respectively, for the Portneuf and Mastigouche/Saint‐Maurice hatchery strains sources). All samples were preserved in ethanol 95% until DNA extraction.

**Table 1:** Information on the 29 selected lakes and their associated domestic strains included in this study on Brook Charr in Québec, Canada

Reserve	Lake	Initial stocking	Final stocking	Genetic sampling	Sample size (*n*)	Number of genotyped individuals	Domestic strain	ADMIXTURE (*q*‐domestic ± *SD*)
Mastigouche	Abénakis (ABE)	1977	2006	2014	22	21	TM	0.135 ± 0.235
Mastigouche	Arbout (ARB)	1973	1980	2014	30	28	TM	0.042 ± 0.182
Mastigouche	Chamberlain (CHA)	1972	2006	2014	23	20	TM	0.107 ± 0.113
Mastigouche	Cougouar (COU)	1975	1995	2014	30	29	TM	0.046 ± 0.078
Mastigouche	Deux‐Étapes (DET)	1972	2012	2014	30	28	TM	0.046 ± 0.081
Mastigouche	Gélinotte (GEL)	1992	2012	2014	30	25	TM	0.060 ± 0.135
Mastigouche	Grignon (GRI)	1977	2013	2014	26	24	TM	0.037 ± 0.072
Mastigouche	Jones (JON)	1975	1989	2014	30	26	TM	0.098 ± 0.240
Mastigouche	Ledoux (LED)	1971	1989	2014	30	27	TM	0.017 ± 0.031
Mastigouche	Lemay (LEM)	1974	1980	2014	30	28	TM	0.018 ± 0.041
Portneuf	Amanites (AMA)	1979	2012	2014	30	29	JC	0.061 ± 0.136
Portneuf	Caribou (CAR)	2008	2013	2014	25	23	JC	0.196 ± 0.238
Portneuf	Daphnies (DAP)	1998	2012	2014	28	27	JC	0.138 ± 0.302
Portneuf	Duhamel (DUH)	1969	2009	2014	23	21	JC	0.013 ± 0.030
Portneuf	Méthot (MET)	1980	2013	2014	30	29	JC	0.312 ± 0.328
Saint‐Maurice	Brown (BRO)	1966	1975	2015	40	28	TM	0.069 ± 0.099
Saint‐Maurice	Brulȏt (BRU)	1970	1972	2015	33	25	TM	0.044 ± 0.076
Saint‐Maurice	Corbeil (COR)	1969	1971	2015	30	23	TM	0.033 ± 0.067
Saint‐Maurice	Gaspard (GAS)	1970	1971	2015	34	31	TM	0.061 ± 0.101
Saint‐Maurice	Maringouins (MAR)	1970	1990	2015	30	26	TM	0.052 ± 0.092
Saint‐Maurice	Melchior (MEL)	1970	1971	2015	30	27	TM	0.045 ± 0.066
Saint‐Maurice	Milord (MIL)	1969	2005	2015	30	25	TM	0.098 ± 0.123
Saint‐Maurice	Perdu (PER)	1964	1990	2015	30	24	TM	0.154 ± 0.175
Saint‐Maurice	Porc‐Épic (POE)	1969	1978	2015	31	26	TM	0.012 ± 0.022
Saint‐Maurice	Portage (POR)	1979	1990	2015	30	27	TM	0.019 ± 0.042
Saint‐Maurice	Soucis (SOU)	1976	1976	2015	31	22	TM	0.151 ± 0.201
Saint‐Maurice	Tempȇte (TEM)	1964	2009	2015	21	18	TM	0.138 ± 0.162
Saint‐Maurice	À la Truite (TRU)	1968	1982	2015	30	24	TM	0.090 ± 0.122
Saint‐Maurice	Vierge (VIE)	1967	1968	2015	45	29	TM	0.001 ± 0.008

Domestic strains TM (*Truites de la Mauricie*) and JC (*Pisciculture Jacques‐Cartier*) represent the main source of the Brook Charr stocked in the selected lakes. The *q*‐domestic values represent the mean membership to domestic strains for each population obtained with the software ADMIXTURE.

### 2.2 Environmental and stocking intensity data

Two types of variables were selected in this study: environmental and stocking intensity variables (see Table 2 for a detailed description and Table S1 in Supporting information for the parameters values for each lake). Firstly, the selection of five environmental parameters with a putative effect on admixture proportion was based on previous studies (Marie et al., 2012; Harbicht, Alshamlih et al., 2014) and on current knowledge of factors influencing physiological conditions of Brook Charr (Power, 1980; Warren, Mineau, Ward, & Kraft, 2010). Thus, data for surface area (ha) and maximum depth (m) were provided by the *Ministère des Forêts, de la Faune et des Parcs* (MFFP*)*. Temperature (^°^C), dissolved oxygen (mg/L), and pH were measured at 1 m below the water surface at the deepest point of each lake. Temperature and dissolved oxygen data were collected with a multiprobe (Seabird, SBE 19plus SeaCat CTD Profiler), and the pH values were obtained using a phTestr 20 (Eutech Instruments). Physico‐chemical parameters were measured twice before the breeding period (June and end of July) in summer 2014 or 2015 and were averaged for each lake, except for pH data for lakes sampled in 2015 that were collected only once during the summer. Secondly, our stocking intensity variables were all determined from data provided by the MFFP and the *Société d’Établissement de Plein Air du Québec* (SEPAQ) and included (i) the number of stocking events, (ii) the quantity of stocked fish per stocking event, and (iii) the mean number of fish stocked per stocking event (Table 2). We also included a time variable represented by the number of years since the mean year over all stocking events (Table 2).

**Table 2:** Description of the environmental and stocking intensity variables used to build models in this study on Brook Charr in Québec, Canada

Parameters	Description	Minimum	Maximum	Median
Environmental factors				
LakeSize	Lake surface area in nectar (ha)	5	273	10
Depth	Lake maximum depth in meter (m)	2.56	29.26	10.85
MeanTemp	Mean temperature of the lake water for the summer of genetic sampling (°C)	16.90	24.52	19.92
MeanO2	Mean concentration of dissolve oxygen in the lake water for the summer of genetic sampling (mg/L)	4.76	8.10	6.17
MeanpH	Mean pH of lake water for the summer of genetic sampling	6.03	7.6	6.77
Stocking intensity				
TotalHa	Total number of fish stocked per hectar	114.47	4660.5	1072.58
NbStockEv	Number of stocking events that occured in the lake	1	38	9
MeanFishStock	Number of fish stocked per stocking event	259	750000	1637.5
SinceMeanYear	Number of years between the genetic sampling and the mean year of stocking	3.45	47.5	31.75

### 2.3 DNA extraction and sequencing

Total DNA was extracted from adipose fin tissue (5 mm^2^) using a slightly modified version of Aljanabi and Martinez (1997) salt extraction protocol. Sample concentration and quality were checked using 1% agarose gel and a NanoDrop 2000 spectrophotometer (Thermo Scientific). DNA quantification was completed using the PicoGreen assay (Fluoroskan, Ascent FL; Thermo Labsystems). Genomic DNA was normalized to obtain 20 ng/μl in 10 μl (200 ng) for each individual. The libraries were created accordingly to Mascher, Wu, St Amand, Stein, and Poland (2013) protocol. Namely, in each sample, a digest buffer (NEB4) and two restriction enzymes (*PstI* and *MspI*) were added. After a two‐hour incubation period at 37°C, enzymes were inactivated by a 20‐min incubation period at 65°C. Then, the ligation of two adaptors was performed using a ligation master mix followed by the addition of T4 ligase and completed for each sample at 22°C for 2 hr. Enzymes were again inactivated by a 20‐min incubation period at 65°C. Finally, samples were pooled in 48‐plex and QIAquick PCR purification kit was used to clean and purified the DNA. After library PCR amplification, sequencing was performed on the Ion Torrent Proton P1v2 chip. Subsequently, FastQC (http://www.bioinformatics.babraham.ac.uk/projects/fastqc/) was used to check raw reads for overall quality and presence of adapters. All the bioinformatic steps, options, and software versions employed in the subsequent GBS pipeline are detailed in Table S2 in Supporting Information. Briefly, we used cutadapt v1.8.1 (Martin 2011) to remove the adapter from raw sequences and STACKS v1.40 *process_radtags* to demultiplex the samples and do the quality trimming (Catchen, Hohenlohe, Bassham, Amores, & Cresko, 2013). Sequence reads were aligned on the rainbow trout (*Oncorhynchus mykiss*) reference genome (Berthelot et al., 2014) with GSnap v9 (Wu & Nacu, 2010). Then, *pstacks* was performed to extract the stacks aligned to the reference genome and to identify SNPs at each locus. *Cstacks* was used to build a reference catalog with all loci identified across all the individuals. Loci from each individual were then matched against the catalog to determine the allelic state in each individual (*sstacks*). Thereafter, the module *populations* were run independently for each lake with the domestic strain used for stocking. Hence, SNPs were defined and called for each lake population combined with its associated domestic source, for a total of 29 different pairs. For each dataset, only individuals with less than 20% of missing genotypes were considered for the subsequent analysis and the R package *stackr* (Gosselin & Bernatchez, 2016) was used to remove loci with more than two alleles. Each output file was also filtered using custom script to retain high‐quality SNPs (available at https://github.com/enormandeau/stacks_workflow). Low variant loci were removed (minor allele frequency <0.10), and only a single SNP per locus was kept to avoid linkage disequilibrium bias (see Table S2 in Supporting information for details about every step).

### 2.4 Estimation of the admixture proportion

First, a random forest method implemented in the package *stackr* (Gosselin & Bernatchez, 2016) in R was used with default arguments to impute missing genotype data by population. Then, the degree of admixture between wild and domestic fish in each lake was assessed using the fast model‐based estimation of ancestry in unrelated individuals implemented in the program ADMIXTURE.v.1.3 (Alexander, Novembre, & Lange, 2009). The software was run on each pair using 2000 bootstraps for potential genetic clusters (*K*) ranging from 1 to 4. *K* values between 2 and 4 were explored to assess whether there was population substructuring within lake (which was not the case, results not shown). Therefore, only the analysis from *K *=* *2 was used for subsequent analyses. Here, our aim was to assess individual ancestry within each wild population to quantify a mean admixture value for each lake representing the extent of domestic introgression. Hence, the proportion of each individual genotype assigned to the domestic cluster (*q*) was averaged for each lake (*q*‐domestic). We also used the function *snmf* implemented in the R package *LEA* as a comparative approach (Frichot, Mathieu, Trouillon, Bouchard, & François, 2014). This method uses non‐negative matrix factorization algorithms and computes least‐squares estimates of ancestry coefficients (Frichot et al., 2014). As the two methods provided similar results (see Supporting information, Fig. S1), only ADMIXTURE results are presented and interpreted.

### 2.5 Model construction

Potential explanatory variables used to build statistical models were tested for correlations between each of them using a Pearson correlation matrix of pairwise correlation coefficients, which revealed some significant correlations (see Supporting information, Table S3). Consequently, variables were centered on the mean and standardized with the standard deviation in order to test for variance inflection factor (VIF) to ensure the absence of multicollinearity between variables (Legendre & Legendre, 2012). Various cutoff values had been proposed in the literature to identify highly collinear variables of which a VIF < 5 is the strictest according to Legendre and Legendre (2012). In our case, all variables showed a VIF value < 3.14 (data not shown) in models, so they were all kept for subsequent analyses. The process of model construction was undertaken to find the best combination of variables explaining and predicting the mean membership to the domestic population. Three different categories of model were thus created: (i) models including environmental variables only, (ii) models including stocking intensity variables only, and (iii) models where both environmental and stocking intensity predictors were included. A common practice used to perform regression analysis on rates and proportions is to perform a logit transformation on the data and then apply a standard linear regression (Ferrari & Cribari‐neto, 2004). However, we avoided this approach as regression estimates are not interpretable in terms of the mean of the untransformed data (Ferrari & Cribari‐neto, 2004). Therefore, models were built using beta regression implemented in the R package *betareg* (Ferrari & Cribari‐neto, 2004) due to the bounded nature of mean domestic membership (between 0 and 1).

### 2.6 Model selection

A three‐step method was used to identify the most suitable model among all models constructed. First (i), given the relatively small sample size (*n *=* *29) and the high number of predictive variables, the second‐order information criterion AICc was used to find the most likely models. Models within 2 ∆_*i*_ units of the best‐fitted model were identified as the most plausible (Akaike, 1976). Second (ii), a jackknife procedure was used to discriminate between the most likely models based on their predictive robustness to avoid circularity that could result from using the exact same data to build the model and test its predictive capacity. This approach consisted of removing one lake at a time for a given model and trying to predict the mean domestic membership of this lake using the model based on the 28 other observations. The difference between the observed mean domestic membership of a lake (obtained with ADMIXTURE) and the predicted value (obtained with the model when the lake was removed) was computed for the 29 lakes and averaged for each model. In this context, the jackknife approach is used to obtain an unbiased prediction and to minimize the risk of over‐fitting (Abdi & Williams, 2010). It is used to evaluate the actual predictive power of our models by predicting the mean domestic membership for each lake as if this observation was a new one. The smaller this average of difference between observed and predicted values is, the better the model should be at predicting the mean domestic membership. Therefore, a model was considered robust enough and was kept in the list of potentially best‐fitted models when its average of differences between observed and predicted values was below a threshold of 0.05. To our knowledge, there is no literature suggesting a precise threshold for the jackknife approach used for determining the predictive quality of models as it is usually employed to simply compare models between each other (Abdi & Williams, 2010). However, this threshold was chosen as we considered that a maximum average difference between predicted and observed values of mean domestic membership of 5% is stringent enough to identify the models with the best predictive quality. Finally, we compared the adjusted *R*‐squared to select the best model among the ones that satisfied the criterion for steps (i) and (ii).

### 2.7 Resilience capacity after stocking cessation

The best‐fitted model was used to predict the value of mean domestic membership after cessation of stocking for a period of 100 years. Time 0 corresponded to the values of mean domestic membership obtained using ADMIXTURE for each sampled lake. The values of the variables representing the quantity of fish stocked into each lake were not changed to simulate stocking cessation. Only the time variable was modulated by adding subsequently 10 years to the initial values of each lake until reaching a simulated period of 100 years after stocking has stopped. The mean domestic genetic membership for each lake was then recorded for every 10 years to illustrate its evolution through time. We also used a model averaging approach to obtain an average estimate of the time variable among all models. The estimated value obtained using model averaging (−0.40; not shown) was very similar to the estimated value obtained with the best‐fitted model only (−0.44; see Results). Thus, the latter was retained for interpreting results.

## 3 RESULTS

### 3.1 DNA sequencing and genotyping

Raw reads demultiplexing and cleaning resulted in 2.17 billion reads with an average of 2.38 million reads per individual. After filtering, 740 of 862 individuals were kept for further analysis. The assembly with the reference genome of the rainbow trout resulted in a catalog containing 951,209 loci. After filtration and keeping one SNP per locus, 4,579 SNPs were obtained on average per pair of populations (lake individuals × associated domestic strain) with values ranging between 3,750 and 5,163 SNPs (Table 3).

**Table 3:** Number of SNPs remaining after filtration steps for the 29 populations of Brook Charr paired with their associated domestic strain used in this study taking place in Québec, Canada

Reserve	Lake	Domestic strains	After population module (SNPs)	After filtering for loci with more than 2 alleles (SNPs)	After custom filtering (SNPs)	First SNPs per locus kept
Mastigouche	Abénakis (ABE)	TM	284759	96397é	6151	5138
Mastigouche	Arbout (ARB)	TM	296552	98419	5994	5073
Mastigouche	Chamberlain (CHA)	TM	291366	99135	6194	5163
Mastigouche	Cougouar (COU)	TM	297918	98967	5935	5026
Mastigouche	Deux‐Étapes (DET)	TM	301417	98150	5646	4809
Mastigouche	Gélinotte (GEL)	TM	295149	97650	5585	4736
Mastigouche	Grignon (GRI)	TM	298066	99269	5743	4850
Mastigouche	Jones (JON)	TM	291407	96360	5978	4980
Mastigouche	Ledoux (LED)	TM	292153	97791	5549	4675
Mastigouche	Lemay (LEM)	TM	285727	96602	5227	4421
Portneuf	Amanites (AMA)	JC	330263	114990	5628	4846
Portneuf	Caribou (CAR)	JC	332548	116255	5449	4729
Portneuf	Daphnies (DAP)	JC	336816	116131	5575	4828
Portneuf	Duhamel (DUH)	JC	337362	116884	6043	5136
Portneuf	Méthot (MET)	JC	346929	120031	5402	4661
Saint‐Maurice	Brown (BRO)	TM	318864	113456	5683	4689
Saint‐Maurice	Brulôt (BRU)	TM	321773	114659	4961	4102
Saint‐Maurice	Corbeil (COR)	TM	333200	115494	5192	4319
Saint‐Maurice	Gaspard (GAS)	TM	285780	99625	5820	4743
Saint‐Maurice	Maringouins (MAR)	TM	329718	115383	5193	4286
Saint‐Maurice	Melchior (MEL)	TM	311428	110727	5474	4512
Saint‐Maurice	Milord (MIL)	TM	345176	122426	5007	4130
Saint‐Maurice	Perdu (PER)	TM	355382	124537	4822	4003
Saint‐Maurice	Porc‐Épic (POE)	TM	355712	125789	4916	4053
Saint‐Maurice	Portage (POR)	TM	373935	129871	5085	4058
Saint‐Maurice	Soucis (SOU)	TM	337661	119059	4521	3750
Saint‐Maurice	Tempête (TEM)	TM	326046	116146	4867	4026
Saint‐Maurice	Á la Truite (TRU)	TM	283111	99871	6092	4941
Saint‐Maurice	Vierge (VIE)	TM	355839	118522	4969	4125

### 3.2 Estimation of the domestic genetic membership

**Figure 2:**
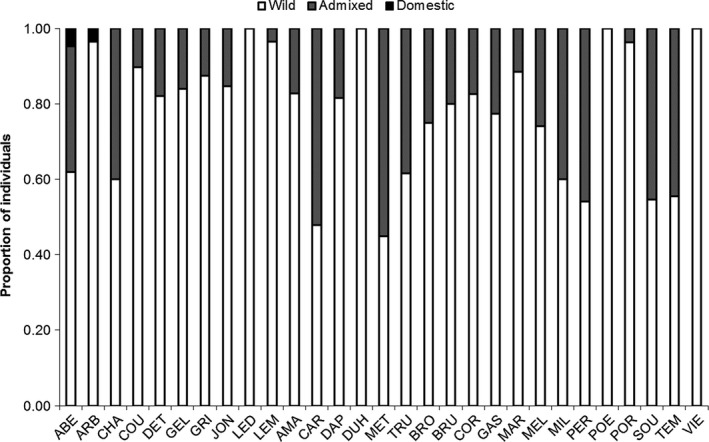
Proportion of individuals assigned to one of the three possible types (wild: *q*‐domestic ≤0.1, admixed: 0.1 < *q*‐domestic <0.9, and domestic: *q*‐domestic ≥0.9) for this study on Brook Charr, in Québec, Canada. Complete names of the populations with the associated abbreviations can be found in Table 1

For all 29 pairs of populations (e.g., each lake and its associated domestic strains), the best clustering solution was always *K *=* *2 (data not shown). The mean domestic memberships within each lake ranged from 0.001 (±0.008) for the lake “VIE” to 0.312 (±0.328) for “MET” (see Table 1 for details and abbreviations). Patterns of individual domestic introgression proportion differed among lakes (Figure 2). “Pure” domestic fish (*q *≥* *0.9) were detected only in two lakes (“ABE” and “ARB”), while “pure” wild fish (q ≤ 0.1) were observed in every lake with a proportion ranging from 0.45 to 1.00. In particular, the lakes “LED,” “DUH,” “POE,” and “VIE” were composed only of pure wild individuals even though these lakes were stocked in the past. The highest proportions of admixed individuals (0.1 < *q *>* *0.9) were found in lakes “CAR” and “MET” with proportion of 0.52 and 0.55, respectively (Figure 2). The barplots of the genomic proportion assigned to the wild or domestic population for each individual of each population are shown in Fig. S2 in Supporting materials.

### 3.3 Selection of the best‐fitted model

**Figure 3:**
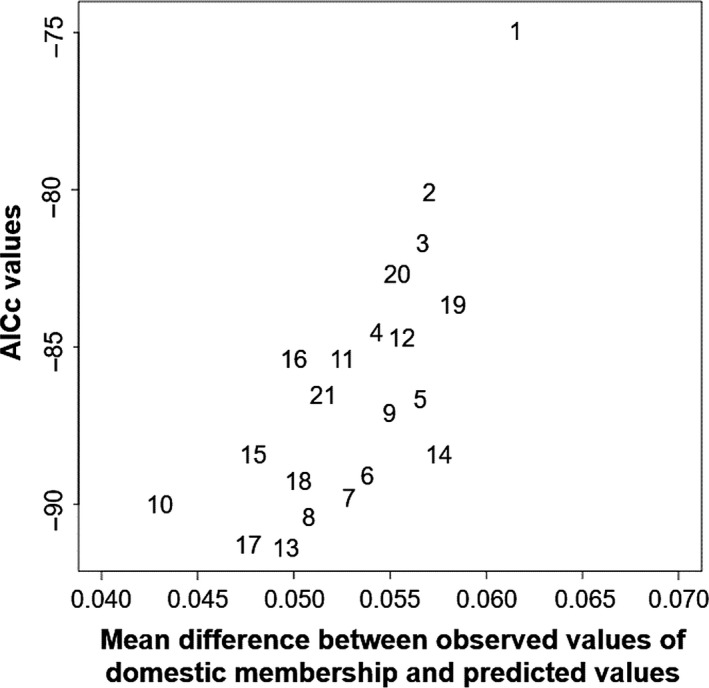
Values of AICc and mean difference between observed values of domestic membership and values predicted by each model using a jackknife approach for this study on Brook Charr in Québec, Canada. Every number corresponds to a model described in Table 4

**Figure 4:**
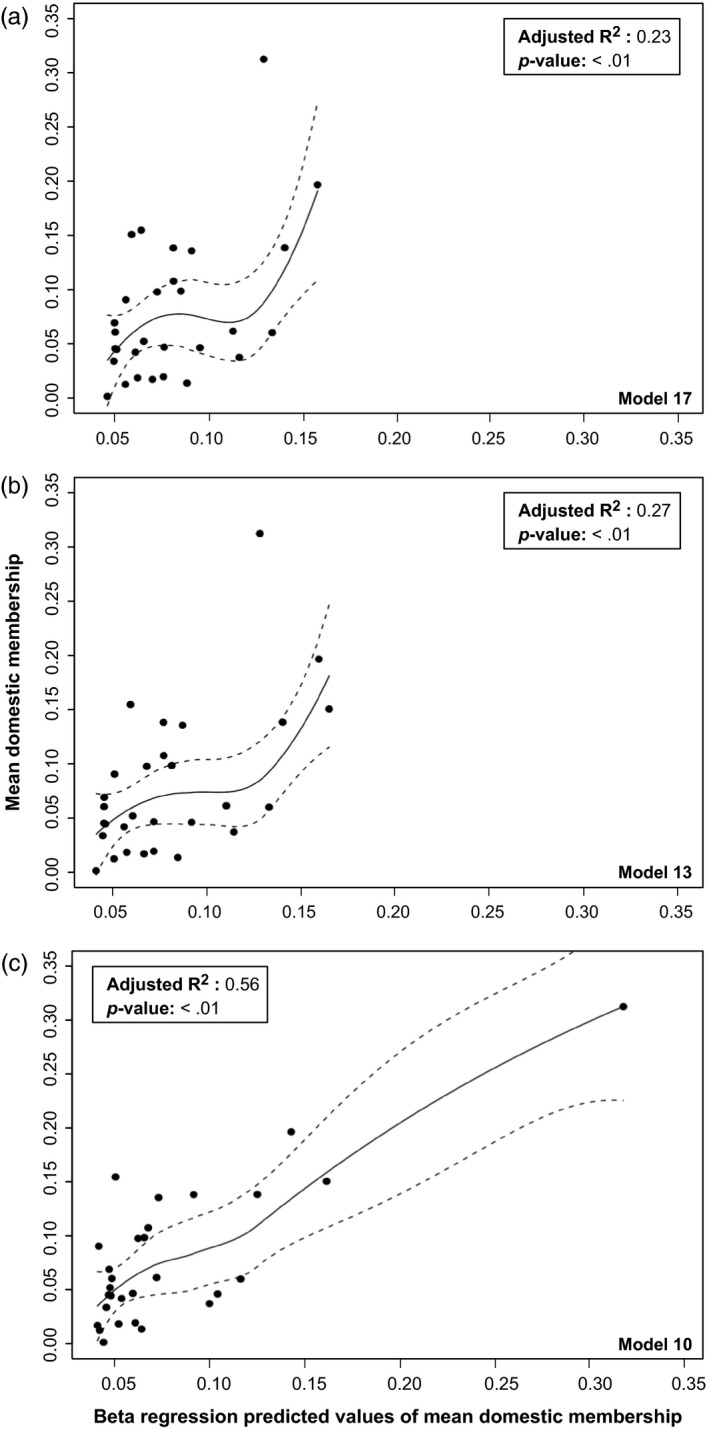
Mean domestic membership observed in each lake as a function of the values of mean domestic membership predicted by the three potentially best‐fitted model for this study on Brook Charr in Québec, Canada. (a) Model 17 = SinceMeanYear, (b) Model 13 = SinceMeanYear + MeanFishStock and (c) Model 10 = SinceMeanYear + NbStockEv × TotalHa + MeanFishStock. Black dots each represent a sampled lake. Complete names and descriptions of the variables included in the models presented here can be found in Table 2

A total of 21 different models were built using stocking intensity variables and environmental factors with a total number of parameters varying between one and five (Table 4). Five models obtained value of AICc ∆_*i*_ under 2: models 13, 17, 8, 10, and 7 (ordered from the smallest to the highest value of ∆_*i*_), indicating that those models are the most plausible (Table 4). Then, the prediction robustness of the models was evaluated using the jackknife procedure (Figure 3). Models 10 (0.043), 15 (0.047), 17 (0.048), 13 (0.049), and 18 (0.049) showed an average difference between predicted and observed values of mean domestic membership <0.05. Thus, only models 10, 13, and 17 were under the acceptance threshold of both tests (Figure 4). The adjusted *R*‐squared values for models 17, 13, and 10 were 0.23, 0.27, and 0.56, respectively (Figure 4). Therefore, because model 10 passed the two first steps of our model selection (AICc and jackknife procedure) and scored the highest adjusted R‐squared value, it was selected as the best‐fitted model (Table 4). Moreover, when these models were run using linear mixed model with lake as a random variable, only model 10 showed a ∆_*i*_ < 2, therefore further suggesting it is the best‐fitted model (data not shown).

**Table 4:** Beta regression models built with stocking intensity variables and environmental factors to explain the observed values of domestic membership in this study on Brook Charr in Québec, Canada

Model	Variables	*k*	*df*	Estimate (±*SE*)	*p*‐Value	Adj. *R* ^2^	Model *p*‐Value	AICc	△_*i*_	W_i_
13	SinceMeanYear	2	4	−0.424 ± 0.124	.0006	0.27	.000247	−91.45	0.00	0.24
	MeanFishStock			0.231 ± 0.102	.0239					
17	SinceMeanYear	1	3	−0.387 ± 0.126	.0021	0.23	.000255	−91.17	0.28	0.17
8	TotalHa	2	4	0.177 ± 0.120	.1423	0.35	.000253	−90.27	1.19	0.13
	SinceMeanYear			−0.368 ± 0.127	.0038					
10	TotalHa	5	4	−0.019 ± 0.161	.2170	0.56	.000219	−89.90	1.55	0.09
	NbStockEv			−0.065 ± 0.178	.1719					
	MeanFishStock			0.301 ± 0.110	.0063					
	TotalHa:NbStockEv			0.216 ± 0.084	.0096					
	SinceMeanYear			−0.440 ± 0.131	.0008					
7	SinceMeanYear	3	5	−0.411 ± 0.126	.0012	0.37	.000247	−89.76	1.70	0.10
	TotalHa			0.148 ± 0.120	.2207					
	MeanFishStock			0.200 ± 0.104	.0541					
18	SinceMeanYear	3	5	−0.402 ± 0.132	.0022	0.35	.000246	−89.42	2.04	0.09
	MeanFishStock			0.241 ± 0.101	.0167					
	MeanTemp			0.118 ± 0.121	.3287					
6	SinceMeanYear	3	5	−0.374 ± 0.143	.0089	0.31	.000247	−89.17	2.28	0.08
	MeanFishStock			0.249 ± 0.103	.0159					
	NbStockEv			0.114 ± 0.138	.4090					
15	NbStockEv:TotalHa	4	6	0.183 ± 0.086	.0342	0.47	.000247	−89.17	2.28	0.08
	NbStockEv			−0.114 ± 0.185	.1874					
	TotalHa			0.082 ± 0.149	.5176					
	SinceMeanYear			−0.407 ± 0.139	.0035					
14	SinceMeanYear:TotalHa	4	6	−0.182 ± 0.122	.1368	0.45	.000238	−88.50	2.95	0.06
	SinceMeanYear			−0.474 ± 0.131	.1037					
	TotalHa			0.053 ± 0.150	.0335					
	MeanFishStock			0.267 ± 0.113	.0183					
9	MeanFishStock	2	4	0.233 ± 0.111	.0361	0.20	.000257	−87.08	4.37	0.03
	NbStockEv			0.299 ± 0.126	.0177					
5	TotalHa	4	6	0.127 ± 0.145	.3820	0.37	.000247	−86.83	4.62	0.02
	SinceMeanYear			−0.377 ± 0.144	.0086					
	MeanFishStock			0.216 ± 0.112	.0534					
	NbStockEv			0.075 ± 0.163	.6447					
21	SinceMeanYear	4	6	−0.414 ± 0.136	.0024	0.31	.000245	−86.45	5.00	0.02
	MeanFishStock			0.167 ± 0.157	.2856					
	LakeSize			0.110 ± 0.185	.5505					
	MeanTemp			0.112 ± 0.121	.3540					
16	NbStockEv:TotalHa	4	6	0.169 ± 0.090	.0576	0.41	.00024	−85.73	5.73	0.01
	NbStockEv			0.186 ± 0.163	.9581					
	TotalHa			0.047 ± 0.172	.5166					
	MeanFishStock			0.260 ± 0.121	.0323					
11	TotalHa:NbStockEv	3	5	0.140 ± 0.090	.8760	0.35	.000251	−85.67	5.78	0.01
	TotalHa			0.130 ± 0.157	.1210					
	NbStockEv			0.140 ± 0.164	.8730					
12	TotalHa	2	6	0.233 ± 0.132	.0787	0.06	.000265	−84.85	6.61	0.01
	MeanFishStock			0.118 ± 0.113	.2950					
4	MeanTemp	1	3	0.190 ± 0.138	.1695	0.19	.000276	−84.70	6.76	0.01
	TotalHa			0.146 ± 0.147	.3197					
19	SinceMeanYear	5	7	−0.395 ± 0.147	.0072	0.35	.000245	−83.88	7.57	0.01
	MeanFishStock			0.095 ± 0.170	.5776					
	NbStockEv			0.066 ± 0.163	.6855					
	LakeSize			0.174 ± 0.185	.3452					
20	TotalHa	3	5	0.229 ± 0.126	.0679	0.32	.000263	−82.82	8.63	0.00
	MeanTemp			0.202 ± 0.128	.1147					
	MeanO2			0.065 ± 0.138	.6386					
3	Depth	2	4	0.091 ± 0.163	.6160	−0.05	.000273	−82.03	9.42	0.00
	LakeSize			0.077 ± 0.169	.6500					
2	MeanpH	3	5	0.072 ± 0.161	.6297	0.12	.000274	−80.29	11.16	0.00
	MeanO2			0.141 ± 0.144	.2971					
	MeanTemp			0.205 ± 0.135	.1284					
1	MeanpH	5	7	−0.049 ± 0.181	.7843	0.07	.000272	−74.68	16.78	0.00
	MeanO2			0.193 ± 0.148	.1945					
	MeanTemp			0.251 ± 0.141	.0746					
	Depth			−0.094 ± 0.199	.6365					
	LakeSize			0.246 ± 0.218	.2589					

The number of parameters in the models (*k*) includes the intercepts, and df represents the number of degree of freedom. ∆_*i*_ corresponds to the AICc delta and models within 2 ∆_*i*_ units of the best‐fitted model (∆_*i*_ = 0.00) are the most plausible. *W*_i_ is the AICc weight of each model. The values of the estimate are based on the centered and standardized values of the parameters. Models are classified from the smallest to the biggest values of AICc. The complete names of the variables presented here can be found in Table 2.

### 3.4 Composition of the most plausible models

Variable “*SinceMeanYear”* (*i.e.,* number of years since the mean year of stocking) was retained in the five best‐fitted models, with estimates ranging between −0.37 and −0.44 (all *p*‐values < .01), indicating its negative relationship with the mean domestic membership. This variable alone explained 23% of the variation observed in mean domestic membership (see Table 4; model 17). In addition, a plot showing the observed mean domestic membership as a function of the variable “*SinceMeanYear”* is presented in supplementary material (Fig. S4). Model 10 was composed of four stocking intensity variables including a positive interaction term between “*TotalHa”* (*i.e.,* total number of fish stocked per ha) and “*NbStockEv”* (*i.e*., number of stocking events). This interaction suggests that, independently, these two variables did not influence mean domestic membership but they did have a positive interactive effect on mean domestic membership when multiplied together. For example, if the number of stocking events is low, the effect of the total number of fish stock/ha will be less important on mean domestic membership. Thus, the higher were the number of stocking events and the total number of fish stocked/ha, the higher was the increase in mean domestic membership. “*MeanFishStock”* (*i.e.,* mean number of fish stocked per stocking event) was also present in models 10 and 13 and was positively related to mean domestic membership estimates (estimates = 0.301 and 0.231, respectively; Table 4). No environmental factors were retained in the most explicative models. However, “*MeanTemp”* (*i.e.,* mean lake temperature) was included in a model having a ∆_*i*_ of 2.04 (model 18) although its predictive contribution to this model was not significant (but see Fig. S3 in Supporting information). The other models including environmental factors were classified as less plausible with ∆_*i*_ **> 4 (Table 4).

### 3.5 Prediction of domestic genetic membership after stocking cessation

**Figure 5:**
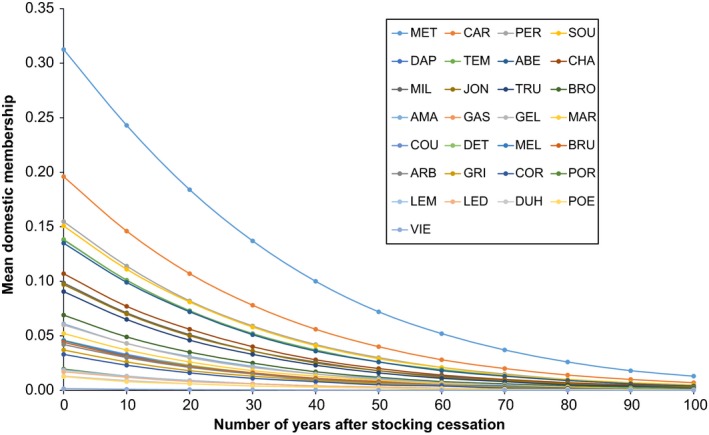
Mean domestic membership as a function of the number of years past after stocking cessation. Values of mean domestic membership for the next 100 years were obtained using model 10 (SinceMeanYear + NbStockEv × TotalHa + MeanFishStock, see Table 2 for the complete names and descriptions of the variables). At time 0, the values showed were obtained with ADMIXTURE for each sampled lake. Complete names of the populations showed here can be found in Table 1

Because the number of years since the mean year over all stocking events was the most important predictive variable within the best models (Table 4), we further investigated its impact on the mean domestic membership. Using model 10, we illustrated its effect by showing for each lake how the mean domestic membership decreased as a function of time (Figure 5). In a scenario where stocking has stopped, the mean domestic membership diminishes with time for each lake until reaching a value near 0, regardless of the initial value of mean domestic membership (Figure 5). For example, lake “MET,” which has a current mean domestic membership of 0.313, would drop to a mean domestic membership value of 0.243 over the first 10 years. Based on these predictions, it would take 40 years for lake “MET” to reach a mean domestic membership of 0.10 (Figure 5).

## 4 DISCUSSION

The main goals of this study were to investigate the extent of introgressive hybridization between wild and domestic populations of Brook Charr using a genomewide approach, to build a model explaining the variation observed across sampled lakes, and to use the best‐fitted model to estimate the resilience potential of wild populations. We thus compared several models based on stocking intensity and environmental variables to select the best model predicting the mean domestic membership of stocked Brook Charr populations. We selected three potential candidate models, including one (model 10) that explained 56% of the variation observed. Interestingly, our most plausible models included only stocking intensity variables, thus suggesting a more limited role for environmental variables as predictors of introgression in this set of lakes. Finally, our results also showed that time since stocking is an important variable predicting the mean domestic membership of a population, as domestic alleles tend to disappear from wild populations when stocking is stopped.

### 4.1 Level of mean domestic membership

Values of mean domestic membership of Brook Charr found in this study were relatively low compared to values reported by previous studies conducted on the same stocked lakes but using 10 microsatellites (“AMA” = 0.520 ± 0.463, “CHA” = 0.385 ± 0.236, “GEL” = 0.777 ± 0.227, and “DET” = 0.350 ± 0.272 in Marie et al., 2010) and 231 SNPs (“AMA” = 0.688 ± 0.413 in Lamaze et al., 2012). Here, none of the 29 sampled lakes showed a mean domestic membership higher than 35%, even when highly stocked. Differences among studies could be explained by several factors. First, Lamaze et al. (2012) and Marie et al. (2010) used the same sampled individuals for their analysis and many more “pure” domestic Brook Charr were captured during their sampling in some lakes, thus inflating the proportion of domestic background in those lakes. For example, Lamaze et al. (2012) identified 33% of “pure” domestic fish in lake “AMA” while our sampling contained no “pure” domestic fish for this population. The fact that several “pure” domestic fish were captured during their sampling could simply be explained by the timing of sampling. For instance, if sampling was conducted just after a stocking event performed during the same summer, the chance of catching “pure” domestic individual would be much higher. In our case, sampling was always performed before a stocking event or in lakes where no stocking event occurred during the summer of sampling. Furthermore, wildlife reserve managers estimate that only 15 to 20% of domestic fish stocked during the spring survive until the next spring because of both fishing and overwintering mortality (*MFFP*, personal communication). This could also explain the low number of domestic fish in our samples. Finally, our study was based on a much higher number of markers than previous ones, thus providing a more complete genomewide coverage of the extent of admixture between populations, given that levels of introgression may vary across the genome, including in salmonid fishes (Ozerov et al., 2016). Therefore, genotyping an average of 4579 SNPs per pair of populations (lake × associated domestic strain) in this study may have resulted in a more precise and realistic estimation of the admixture level. As such, we are confident that our results provide reliable estimates of the “true” proportion of admixture in this system.

Furthermore, the relatively low level of introgression observed in our study suggests that the introgression of domestic alleles into wild populations is relatively weak, which is consistent with previous observations showing that domestic reared salmonids fish reproduce very poorly in the wild (Araki et al., 2008). For example, an experimental study conducted by Lachance and Magnan (1990) compared the recovery rates after 2 years of wild, hybrid, and domestic strains of Brook Charr in six small oligotrophic lakes in Québec. They showed that wild strain performed better than domestic strain after stocking (with the hybrid strain having an intermediate performance). Various hypotheses have been suggested to explain the low survival of domestic strains under natural conditions such as a low adaptability of individuals to the available food resources causing starvation (Ersbak & Haase, 1983), a high susceptibility to predation (Vincent, 1960), and low resistance to stress (Vincent, 1960).

### 4.2 Key variables explaining introgression level

Our most plausible models have highlighted the importance of four variables linked with stocking intensity: (i) the number of years since the mean year of stocking, (ii) the interaction between the total number of fish stocked per ha, (iii) the number of stocking events, and finally (iv) the mean number of fish stocked per stocking event. The number of years since the mean year of stocking was present and significant in all the best‐fitted models, emphasizing its importance in predicting mean domestic membership in stocked lakes. More specifically, our results confirm the evidence in this species that the mean domestic membership decreased with an increasing number of years since the mean year of stocking. Valiquette et al. (2014) previously showed that populations of Lake Trout that were not stocked for a longer time tended to have a lower level of admixture. Altogether, these results suggest that, at least in some circumstances, populations identified as being “polluted” by domestic alleles may eventually be considered “wild” again, given sufficient time since the stocking cessation.

In addition, the best‐fitted model (model 10) was composed of an interaction between the total number of fish stocked/ha and the number of stocking events, which suggest an increase of the mean domestic membership of a population *via* a high number of stocking events associated with a high number of fish stocked per ha in a lake. Arguably, adding of this interaction to model 10 mainly contributed to improve the mean domestic membership prediction of lake “MET.” The models 13 and 17 (the two other potentially best‐fitted models) did not retain this interaction as an explicative term and did not include the lake “MET” within 95% IC boundaries as the model 10 did. Indeed, lake “MET,” which has the highest mean domestic membership, was also unique in having a very high number of stocking events (38) and a high density of stocked fish (4660,5 fish/ha; which are the two variables composing the interaction) and may thus have influence this result. However, including this interaction term allowed the defining of a robust model (model 10) based on the results of the jackknife approach. We are confident that the presence of this interaction between the total number of fish stocked/ha and the number of stocking events could help the performance of our best‐fitted model at predicting lake with high level of mean domestic membership. Furthermore, our results are similar to those from previous studies that reported positive effects of the total number of fish stocked per ha and/or the number of stocking events on observed admixture levels (Eldridge & Naish, 2007; Finnegan & Stevens, 2008; Marie et al., 2010, 2012; Perrier et al., 2012; Valiquette et al., 2014).

Finally, the mean number of fish stocked per stocking event had a modest yet significant positive effect on the mean domestic membership. To our knowledge, this is the first report of a link between the admixture level and this variable. Nevertheless, adding a high absolute number of fish in a lake should be expected to increase the probability of hybridization between wild and domestic fish, by increasing the probabilities of reproduction between wild and stocked fish. For instance, this variable was particularly relevant to predict admixture of the lake “SOU.” The stocking history of this lake is distinct from other lakes in our sample as only one stocking event was performed but with 750,000 domestic fish being introduced. This very high number of domestic individuals added in the lake could explain its relatively high domestic membership compared to the other sampled lakes, even if no stocking occurred in this lake for the last 38 years.

### 4.3 Environmental variables vs. introgression levels

Previous studies suggested that environmental variables (*i.e.,* temperature, pH, dissolved oxygen, lake size, and depth) played a role in explaining variation in the extant of introgression among stocked Brook Charr populations (Marie et al., 2012; Harbicht, Alshamlih et al., 2014). Also, it has been long argued that factors reducing habitat quality may enhance hybridization between wild populations and their domestic congeners (Rhymer & Simberloff, 1996). For example, when lake water temperature increases during summer, Brook Charr could be more constrained in suitable thermal habitat for them. Thus, fish could gather in the few thermal refuges where water stays colder and therefore contacts between the domestic and wild populations could be enhanced (Biro, 1998; Marie et al., 2012). However, in our study, none of the environmental variables were included in the more plausible models predicting mean domestic membership and neither did they improve significantly the prediction capability of the selected best‐fitted models. Yet, when we plotted mean domestic membership with each environmental parameter separately, we observed that the mean temperature significantly explained part of the variation in mean domestic membership. That is, mean domestic membership tended to increase slightly as the temperature of the lakes increased. Moreover, the mean temperature variable was also present (but not significant) in a moderately plausible model (2 < ∆_i_ < 4). Admittedly, however, we could only collect temperature information at two points in time such that further investigation of the environmental variables should be conducted with more detailed data (e.g., exhaustive seasonal temperature profiling) to more firmly investigate the role of such environmental variables on patterns of introgression.

### 4.4 Resilience potential after stocking cessation

Using the model 10 and the number of years since the mean year of stocking as a variable of time, we showed that the level of domestic membership decreases with mean time of stocking in our lakes. Indeed, all lakes showed a decrease of domestic alleles through time according to the best‐fitted model predictions. So, it is possible that combination of domestic alleles which are unfit for the local environment could be purged through time by selective processes in the wild. This implies that, in the same circumstances, the stocking effects could be reversible if wild populations are resilient enough to persist through time as proposed in other studies on salmonids (Hansen & Mensberg, 2009; Perrier et al., 2013; Valiquette et al., 2014). In addition to selection, genetic drift could contribute to purge the exogenous alleles if their frequencies are lower than the wild ones (Frankham et al., 2010). However, it should be noted that none of our lakes showed a mean domestic membership higher than 0.35. It is thus difficult to predict what would happen in lakes with high level of mean domestic membership (ex.: >0.80). Indeed, as domestic alleles would be in higher frequencies than wild ones in such populations, genetic drift could instead fix domestic alleles through stochastic effects (Frankham et al., 2010). Under these circumstances, we may expect a “tug‐of‐war” between natural selection against domestic alleles and genetic drift that could tend to fix the domestic alleles. It is thus possible that in lakes with very high level of introgression, the population may not be able to return to an original state.

### 4.5 Model improvement

While our best‐fitted model selected explained a significant proportion (56%) of the variation observed in introgression level between wild and domestic populations of Brook Charr, other aspects should be considered toward improving our predictive capacity. First, adding new variables could help explaining a higher part of the mean domestic membership variation. For instance, the age/stage of stocked fish could have an impact on introgression level, as older brook trout life stages (fingerlings, yearlings, and adults) have typically a higher survival rate than younger life stage (fry) when stocked in the wild (Kerr, 2000). Furthermore, the time period where stocking occurred could also be important for determining introgression level as fish stocked at the end of spring or beginning of summer are more likely to survive than fish stocked later on during the fall (Kerr, 2000; Harbicht, Alshamlih et al., 2014). Fishing pressure could also have a role to play in introgression level as Harbicht, Alshamlih et al. (2014) suggested that angling intensity was negatively correlated to admixture in Brook Charr populations in Ontario (Canada) as anglers tended to be more efficient at angling domestic fish (Mezzera & Largiadèr, 2001). Unfortunately, the available data for these variables were incomplete and could not be used here. Indeed, model selection cannot take into consideration missing values for the explanatory variables, such that they were eliminated from the final choice of variables. Another important variable missing in our models is the initial wild populations’ effective size before stocking, as no inventory of the selected populations was made before stocking. The outcome of introgression could differ between populations with different initial effective size even though they underwent similar stocking intensity (Hansen, 2002). Thus, reduced effective wild population size is expected to further enhance the effect of stocking as described by Hansen et al. (2009) and Perrier et al. (2012) for brown trout (*Salmo trutta*) and Atlantic salmon (*Salmo salar*), respectively. Consequently, when available, such natural variables are usually taken into consideration in hatchery management plans (Mobrand et al., 2005; Lorenzen, Beveridge, & Mangel, 2012; Baskett, Burgess, & Waples, 2013). Second, adding more sampled lakes could help improving the predictive power of the model. Moreover, to assess more firmly the importance of the interaction between the total density of stocked fish and the number of stocking events and their effect on level of introgression, more lakes with high level of stocking intensity (such as lake “MET”) would be required. Finally, it would also be relevant to test our best‐fitted model by predicting the mean domestic membership of other lakes not included in this study to further assess the predictive capacity of the model.

## 5 PRACTICAL APPLICATIONS FOR MANAGEMENT AND CONSERVATION

The best‐fitted model developed in this study represents the best tool available until now to predict the introgressive hybridization level between wild and domestic populations in any salmonid species. In the specific context of Brook Charr management in Québec, this model could be easily used by wildlife managers by adding the values of the variables composing the best‐fitted model in the equation provided in the associated excel spreadsheet (see Appendix S1). The equation will predict the actual value of mean domestic membership of a population from the values provided for the explanatory variables. Then, by adding years to the variable of time present in the model, it will possible to predict what will be the mean domestic membership of this population through time if stocking is stopped. One management measure that could be applied from this observation would be to determine a threshold at which a lake would be considered to be back to an “original” genetic state. We suggest to use a threshold value of mean domestic membership of 0.10, as fish are individually considered as wild when their domestic background proportion is lower than 0.10 (Marie et al., 2010; Lamaze et al., 2012). Thus, if the mean domestic membership of a population is less than 0.10, it could be interpreted as being genetically similar to a wild state. Using the best‐fitted model, it would also be possible to predict the number of years during which stocking need to be stopped to allow the mean domestic membership to decrease until reaching the selected threshold of 0.10. The principle of “fallow” used in durable agriculture could then be applied to the management of Brook Charr populations for recreative fishing. Thus, some lakes could be kept “stocking‐free” for a certain number of years while other lakes could still be stocked to support more intensive recreative fishing and a rotation of those lakes could be done. More generally, this type of management practice has still not been applied in any salmonid species, at least to our knowledge. Thus, with further improvements and investigations, this simple approach of predicting hybridization level between wild and domestic populations using various explanatory variables and including a variable of time could serve as a model for the conservation and management of other wild populations of salmonids being stocked. This type of solution could lead the way to a more durable exploitation of salmonid species and a more adequate management of stocking.

## DATA ARCHIVING STATEMENT

Data available from the Dryad Digital Repository: https://doi.org/10.5061/dryad.s5qt3.
