# Supplementary material for: Predicting the genetic impact of stocking in Brook Charr (Salvelinus fontinalis) by combining RAD sequencing and modeling of explanatory variables
Source: Evol Appl. 2017 Nov 13;11(5):577–92. doi: 10.1111/eva.12566 (PMC5978948; doi:10.1111/eva.12566)
Supplement: Supplementary file 1 [file EVA-11-577-s001.docx]

**Supporting information**

**Table S1.** Values of selected explanatory variables for each lake sampled for this study on Brook Charr in Québec, Canada. For details description of the variables and their complete names see Table 2 in the main text.

**Table S2.** Detailed methods, options and values for each filter used to identify SNPs for this study on Brook Charr in Québec, Canada.

**Table S3.** Pearson correlation matrix of pairwise correlation coefficients for environmental parameters and stocking intensity variables included in this study on Brook Charr in Québec, Canada. Bold values indicate significant correlation (α = 0.05). * are significant after Bonferroni correction. The description of the variables presented in this table can be found in Table 2 in the main text.


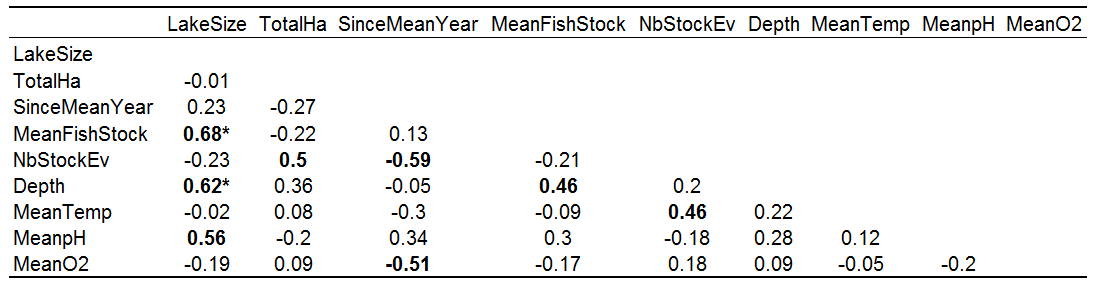


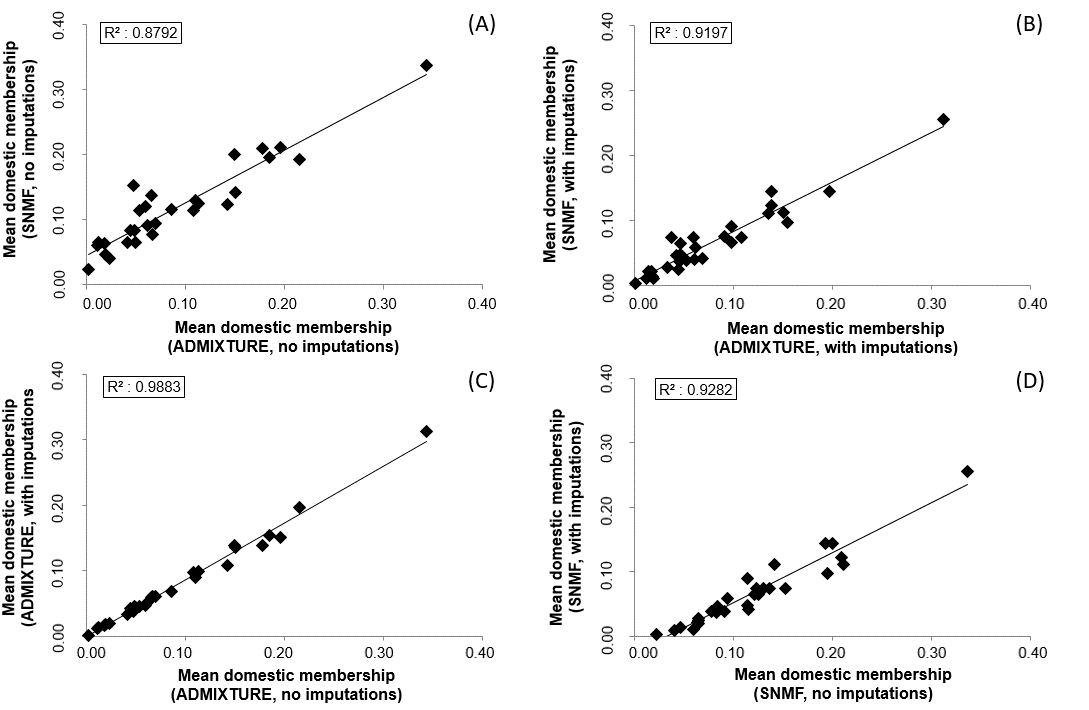


**Figure S1.** Comparisons of the results obtained with the software ADMIXTURE and SNMF ran with or without imputations for this study on Brook Charr in Québec, Canada. (A) Mean domestic membership obtained with SNMF without imputations as a function of the mean domestic membership obtained with ADMIXTURE without imputations. (B) Mean domestic membership obtained with SNMF with imputations as a function of mean domestic membership obtained with ADMIXTURE with imputations. (C) Mean domestic membership obtained with ADMIXTURE with imputations as a function of the mean domestic membership obtained with ADMIXTURE without imputations. (D) Mean domestic membership obtained with SNMF with imputations as a function of mean domestic membership obtained with SNMF without imputations. Black diamonds represent sampled lakes.

**Figure S2.** Bayesian individual clustering results for each lake (classified by wildlife reserve) of this study on Brook Charr in Québec, Canada. Columns represent proportion of membership of each individual to each cluster and rang from 0 to 100%.


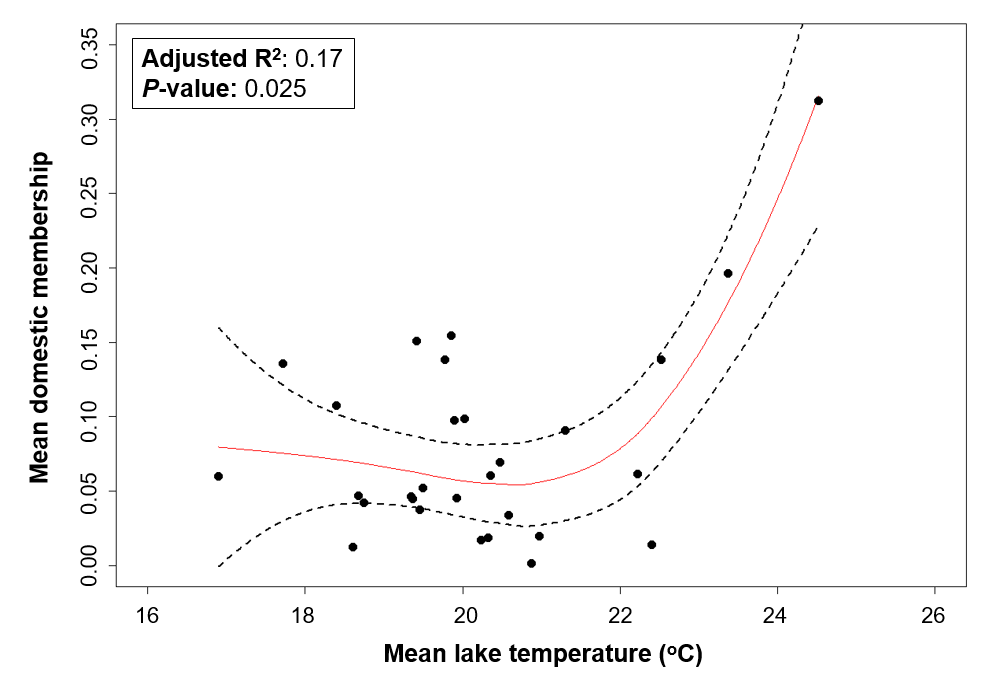


**Figure S3.** Mean domestic membership observed in each lake as a function of the mean temperature for this study on Brook Charr in Québec, Canada.


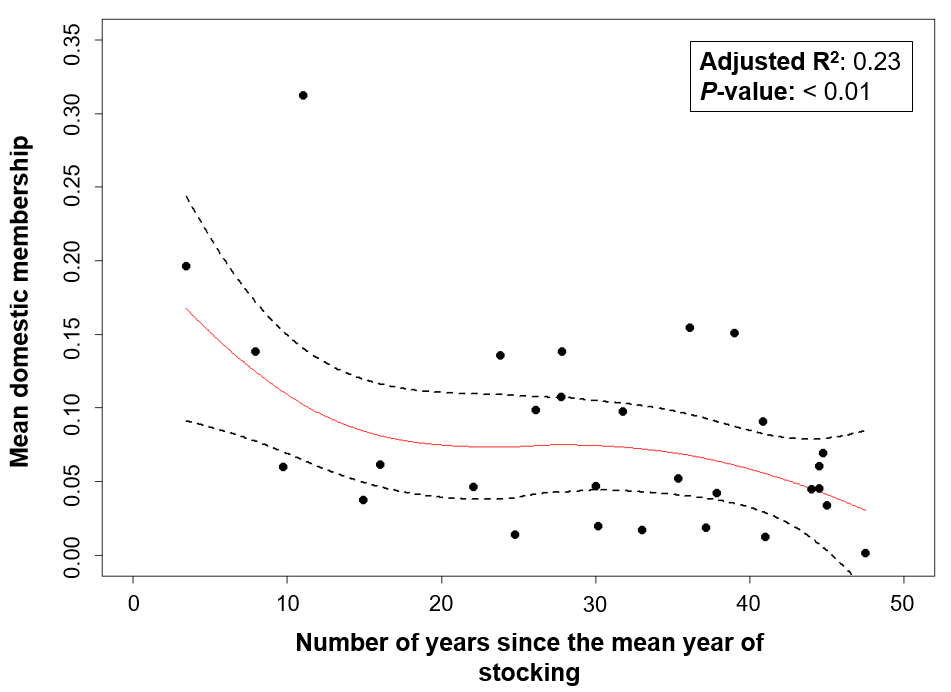


**Figure S4.** Mean domestic membership observed in each lake as a function of the number of years since the mean year of stocking for this study on Brook Charr in Québec, Canada
